# Calibrated CAR Signaling Enables Low-Dose Therapy in Large B-Cell Lymphoma

**DOI:** 10.21203/rs.3.rs-4619285/v1

**Published:** 2024-07-02

**Authors:** Jae Park, M. Lia Palomba, Karlo Perica, Sean Devlin, Gunjan Shah, Parastoo Dahi, Richard Lin, Gilles Salles, Michael Scordo, Karthik Nath, Yannis Valtis, Alec Lynch, Elizabeth Cathcart, Honglei Zhang, Heiko Schöder, Doris Leithner, Kelly Liotta, Alina Yu, Kelsey Stocker, Jia Li, Agnish Dey, Leopold Sellner, Reshma Singh, Varsha Sundaresan, Faye Zhao, Jorge Mansilla-Soto, Changhao He, Joel Meyerson, Kinga Hosszu, Devin McAvoy, Xiuyan Wang, Isabelle Riviere, Michel Sadelain

**Affiliations:** Memorial Sloan Kettering Cancer Center; Memorial Sloan Kettering Cancer Center; Memorial Sloan Kettering Cancer Center; MSKCC; Memorial Sloan Kettering Cancer Center; Memorial Sloan Kettering Cancer Center; Memorial Sloan Kettering Cancer Center; Memorial Sloan Kettering Cancer Center, New York, USA; Memorial Sloan Kettering Cancer Center; Memorial Sloan Kettering Cancer Center; Memorial Sloan Kettering Cancer Center; Memorial Sloan Kettering Cancer Center; Memorial Sloan Ketterning Cancer Center; Memorial Sloan Kettering Cancer Center; Department of Radiology, Memorial Sloan Kettering Cancer Center, New York, NY; Memorial Sloan Kettering Cancer Center; Memorial Sloan Kettering Cancer Center; Memorial Sloan Kettering Cancer Center; Memorial Sloan Kettering Cancer Center; Takeda Development Center Americas, Inc; Takeda Development Center Americas, Inc; Takeda Development Center America Inc.; Takeda Development Center Americas, Inc; Takeda Development Center America Inc.; Takeda Development Center Americas, Inc; Moffitt; Weill Cornell Medical College; Weill Cornell Medical College; Memorial Sloan Kettering Cancer Center; Memorial Sloan Kettering Cancer Center; Memorial Sloan Kettering Cancer Center; Memorial Sloan Kettering Cancer Center; Memorial Sloan Kettering Cancer Center

## Abstract

We designed a CD19-targeted CAR comprising a calibrated signaling module, termed 1XX, that differs from that of conventional CD28/CD3z and 4–1BB/CD3z CARs. Here we report the first-in-human, phase 1 clinical trial of 19(T2)28z-1XX CAR T cells in relapsed/refractory large B-cell lymphoma. We hypothesized that 1XX CAR T cells may be effective at low doses and investigated 4 doubling dose levels starting from 25×10^6^ CAR T cells. The overall response rate (ORR) was 82% and complete response (CR) rate 71% in the entire cohort (n=28) and 88% ORR and 75% CR in 16 patients treated at 25×10^6^. With the median follow-up of 24 months, the 1-year EFS was 61% (95% CI: 45–82%). Overall, grade ≥3 CRS and ICANS rates were low at 4% and 7%. The calibrated potency of the 1XX CAR affords excellent efficacy at low cell doses and may benefit the treatment of other hematological malignancies, solid tumors and autoimmunity.

CD19-directed chimeric antigen receptor (CAR) T-cell therapies have produced impressive anti-tumor efficacy resulting in approval of several products for relapsed or refractory (r/r) large B-cell lymphoma (LBCL)^1–6^. However, clinical outcomes remain suboptimal in LBCL with the current approved CD19 CARs that reported complete response (CR) rates of 40–65% and estimated 1-year progression-free survival rates of 44–46% in the 2nd and ≥ 3rd line settings^1–4^. Furthermore, most CD19 CAR products are associated with significant toxicities such as cytokine release syndrome (CRS) and immune effector-cell associated neurotoxicity syndrome (ICANS)^1–11^. Therefore, there is a need for more potent CAR that can generate higher and more durable responses with minimal toxicity.

Two CAR designs, encompassing either the CD28^1,2,7,12^ or 4–1BB costimulatory signaling domain^3,4^ fused to the CD3z chain, are used in all current commercially approved CAR products and in the majority of investigational studies^13,14^. CD28-based CAR T cells are characterized by rapid expansion and robust effector function but limited persistence and high rates of severe CRS and ICANS^1,2^. We hypothesized that redundancy of CD28 and CD3z signaling accelerates T cell differentiation and promote T cell exhaustion and therefore designed 1XX CARs, which bear inactivating point mutations in the two distal immunoreceptor tyrosine-based activation motifs (ITAMs) and retain one functional proximal ITAM^15^. In preclinical studies, we demonstrated that 1XX CARs displayed extended persistence and could clear tumor at lower doses^15^ than archetypal CD28/CD3z CARs^16^.

Based on these preclinical data, we hypothesized that 1XX CAR T cells would require a lower cell dose to achieve comparable tumor responses and reduce the incidence of severe CRS and ICANS, as those toxicities are associated with high T cell doses^17,18^. To test this hypothesis, we designed the first-in-human phase I clinical trial to identify the lowest effective dose of 19(T2)28z-1XX CAR T cells in patients with r/r LBCL. While CD19 is not a novel target, it remains the quintessential and ideal disease space for a single-arm study to test novel engineered modalities or new receptor designs given the well-established long-term outcome of CD19 CARs in lymphoma and potential applicability to other CD19 + lymphoid malignancies such as acute lymphoblastic leukemia and chronic lymphocytic leukemia where there are larger unmet needs^12,19^. Herein we report the first full clinical outcome data of dose escalation and expansion phase of the study.

## Results

### Study Design and Patient Characteristics

**Figure 1:**
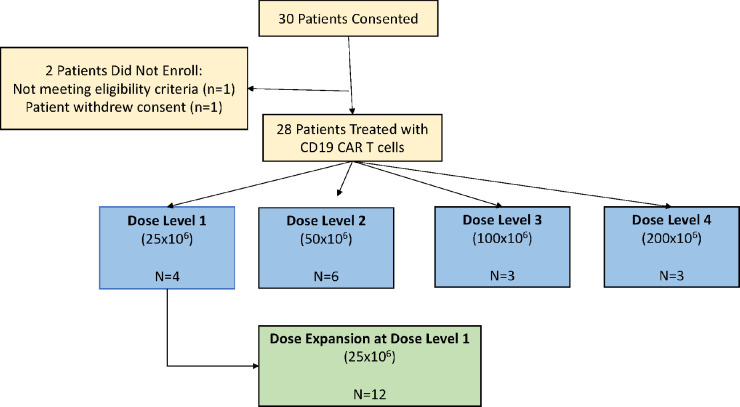
Consort Diagram

From July 2020 to November 2022, a total of 30 patients consented and 28 patients underwent apheresis and received treatment with 19(T2)28z-1XX CAR T cells; one patient did not meet eligibility criteria (no evidence of disease at screening) and one chose alternative treatment (Fig. 1). Sixteen patients were treated in the dose escalation cohort and 12 in the dose expansion cohort. The cell products for all 28 patients were manufactured successfully and the median manufacturing time was 7 days (range, 7–10). The data cutoff date for this analysis was May 30, 2024.

Baseline patient characteristics are summarized in Table 1. The median age of the overall cohort was 65 years (range, 48 to 86), and 13 patients (46%) were 65 years and older. Patients had a histological diagnosis of r/r LBCL (61%), transformed indolent B-cell lymphoma (25%), and high-grade B-cell lymphoma (14%). Twenty-four patients (86%) had primary refractory disease, and 25 (89%) had disease refractory to immediate last line of therapy. Eleven patients (39%) had ≥ 2 prior lines of therapy, and 3 (11%) received prior CD19-directed therapy (tisagenlecleucel, n = 2; tafasitamab, n = 1). Twenty-five (89%) patients received bridging therapy (19 chemotherapy and 6 radiation only). The detailed individual patient and disease characteristics including baseline laboratory and disease burden assessments can be found in **Extended Data** Table 1.

### Safety

All adverse events of any grade in the entire cohort regardless of causality are listed in Table 2. The most frequent all grade adverse events were CRS (grade 1–2: 82%; grade 3: 4%), diarrhea (25%), nausea (21%), and edema (18%). Grade 3 and higher adverse events of any kind were infrequent and included ICANS (7%), CRS (4%), hypotension (4%), generalized edema (4%), sepsis (4%), and atrial fibrillation (4%) (Table 2). One patient experienced dose-limiting toxicity (grade 3 ICANS) at DL2 (50×10^6^), and additional 3 patients were treated in that cohort with no further DLT event.

**Figure 2:**
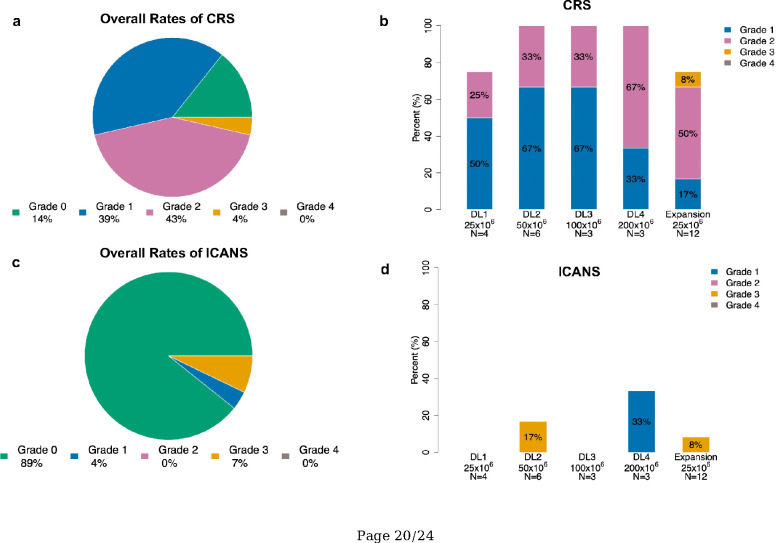
Rates of CRS and ICANS in all cohorts (2A) Overall rate of CRS by grade. (2B) Rate of CRS by grade and by dose level of CAR T cells. (2C) Overall rate of ICANS by grade. (2D) Rate of ICANS by grade and by dose level of CAR T cells. CRS denotes cytokine release syndrome; ICANS denotes immune effector-cell mediated neurotoxicity syndrome; DL denotes dose level.

Twenty-four patients (86%) experienced CRS. All occurrences of CRS were grade 1 (n = 11, 39%) or 2 (n = 12; 43%), except for 1 grade 3 CRS (4%) (Fig. 2a). Severity and occurrences of CRS by dose levels are shown in Fig. 2b. No patient experienced grade 4 or 5 CRS. Median time to CRS onset was 4 days (range, 1–11) and median duration was 4 days (range, 1–9). Seventeen patients (61%) received tocilizumab and 9 (32%) received steroids as treatment for CRS. ICANS was observed in 3 patients: grade 1 (n = 1, 4%) and grade 3 (n = 2, 7%) (Fig. 2c). No patient experienced grade 4 or 5 ICANS. Median time to ICANS onset was 8 days (range, 7–10 days), and median duration was 9 days (range, 3–10). All 3 patients with ICANS received steroids as treatment with resolution of symptoms.

### Efficacy

**Figure 3:**
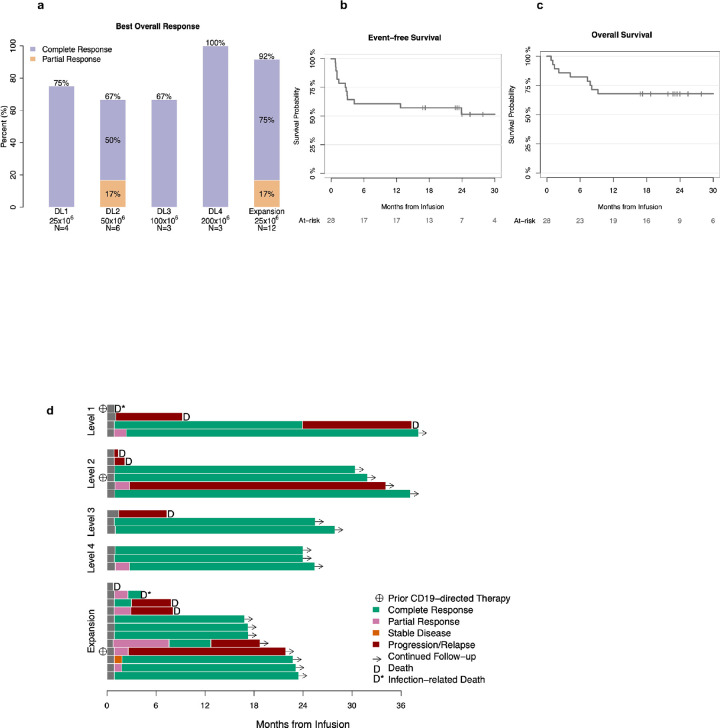
Response of patients and survival rates (3A) Best overall response by dose level. (3B) Event free survival and (3C) Overall survial of the study patients. (3D) Swimmer plot depicting the timing of response, relapse and clinical outcome over time.

In the dose escalation phase, 12 of 16 patients had a response with an ORR of 82%; 11 patients achieved CR (69%) and 1 (6%) achieved partial response (PR) as their best overall response (Fig. 3a). Responses were observed across all dose levels: 3 of 4 patients (75%) in DL1, 4 of 6 (67%) in DL2, 2 of 3 (67%) in DL3 and 3 of 3 (100%) in DL4 (Fig. 3a).

Based on equivalent clinical responses observed across all dose levels (25–200×10^6^) and to test the hypothesis that low dose 1XX CAR will achieve high response rates with low toxicity^15^, we expanded the lowest dose cohort (25×10^6^) and treated 12 additional patients at that dose level. Of the 12 patients, 9 patients achieved CR (75%) and 2 achieved PR (17%) with 92% ORR (Fig. 3a). When assessed in all 16 patients treated at 25×10^6^ CAR T cells during dose escalation (n = 4) and dose expansion cohorts (n = 12), CR was observed in 75% and PR in 13% with an ORR of 88%. Only 1 patient, an 86-year-old woman with primary refractory DLBCL, experienced severe CRS and ICANS that lasted for < 48 hours and ultimately achieved CR.

Of the 3 patients who received prior CD19-directed therapies, 1 patient achieved PR (DL1) and 2 patients CR (DL1 and 2), one of whom is in ongoing CR at 2.5 years post-treatment.

The median follow-up duration among survivors was 24 months (IQR, 22–32). The Kaplan-Meier probability of response duration for at least 3 months was 74% (95% CI, 58 to 94%). The 1-year EFS were 61% (95% CI: 45–82%), and 14 patients remain in continuous CR beyond 12 months (Fig. 3b). Overall survival (OS) at 1-year was 68% (95% CI: 53–88%); the median OS was not reached (Fig. 3c). The individual patient outcomes by treated dose levels are shown in Fig. 3d. There was a total of 10 deaths in the study group: 8 from disease progression and 2 from infections. Seven patients who died of disease progression had refractory disease to CAR T cell therapy (no response or < 3 months ORR) and died at a median of 66 days from T cell infusion (range, 23–281), and one patient had a late relapse at 2 years and died at 37 months from T cell infusion. Of the 2 patients who died of infections, one was a 78-year-old woman with an indwelling nephro-ureteral stent and a history of frequent urinary tract infection who died of sepsis at day 33, and the second an 86-year-old woman who died of shigella enterocolitis 129 days from T cell infusion.

### Correlative Biomarker and Cellular Kinetic Analysis

Since the majority of deaths related to disease progression (7 of 8) occurred in patients refractory to CAR T cell therapy in this study, we examined for disease related factors associated with refractory disease, i.e. < 6 months CR. We found that LDH (p < 0.001) and disease burden as measured by metabolic tumor volume at screening (p = 0.005) significantly correlated with refractory disease and lower EFS (**Extended Data** Tables 2 and 3). There was no difference in EFS between the 25×10^6^ and 50–200×10^6^ cohorts (**Extended Data Table 3**), but notably the low dose cohort (25×10^6^) included a numerically higher proportion of patients with higher tumor volume at screening (84, IQR 26–436 vs. 28, IQR 16–71) although the difference was not statistically significant (**Extended Data Table 4**).

**Figure 4:**
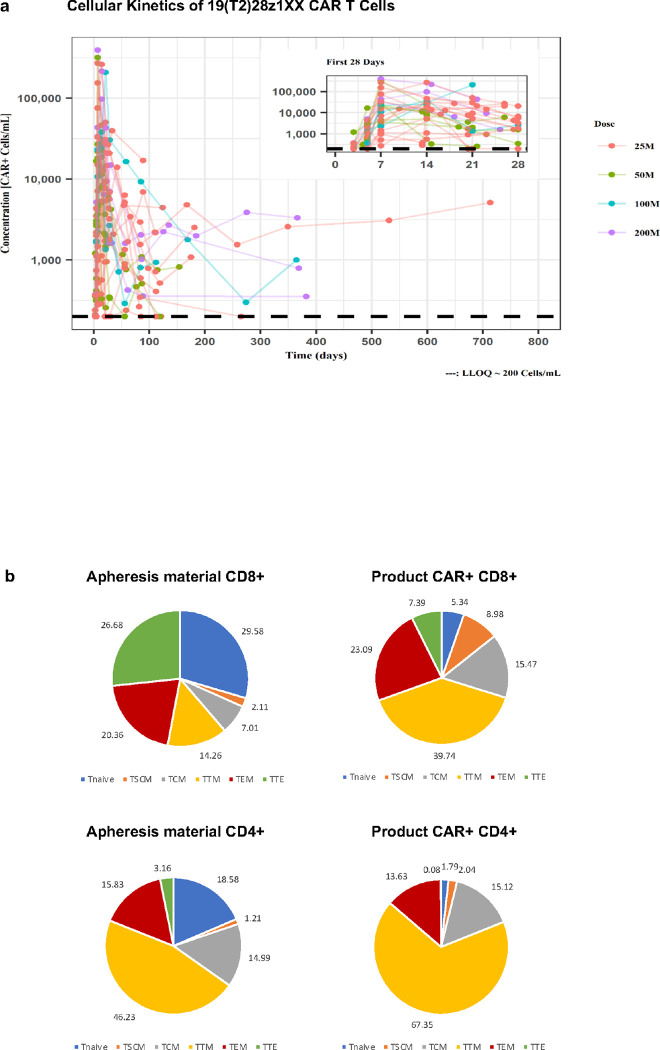
Cellular kinetics and T cell immunophenotypes **(4A)** Individual peripheral blood (PB) cellular kinetic profiles of 19(T2)28z1xx CAR T cells in 27 patients with reportable PB cell concentrations measured by quantitative polymerase chain reaction method. The horizontal line at 200 cells/mL represents the lower limit of quantification of this assay. Each line represents individual subject cellular kinetic curve color coded by dose levels. Inner panel highlights the cellular kinetics within the first 28 days of T cell infusion. Red, green, blue, and magenta represent 25M, 50M, 100M and 200M dose levels. M: Million CAR+ cells.**(4B)** Composition of CD8 and CD4 T cell subsets in the apheresis material and final infusion product represented as pie charts with average percentages. T_naive_: naïve T cell; T_SCM_: stem cell memory T cell; T_CM_: central memory T cell; T_TM_: transitional memory T cell; T_EM_: effector memory T cell; T_TE_: terminal effector T cell

Robust T cell expansion was observed across all dose levels with no difference in peak CAR T cell expansion (C_max_) (Fig. 4a **and Extended Data** Fig. 1). We observed CAR T cells persisting beyond one year in 5 patients in ongoing CR including a patient treated at DL1 with detectable CAR T cells beyond 2 years (Fig. 4a). There was a trend toward higher exposure to CAR T cells during the first 28 days of infusion (AUC_0 – 28_) with increasing infused CAR T cell doses, but no significant correlation between C_max_ or AUC_0 – 28_ and response (**Extended Data** Fig. 1) and no correlation between C_max_ and refractory disease (**Extended Data** Table 2). Patients with higher C_max_ and AUC_0 – 28_ had an increased likelihood of experiencing > grade 1 CRS (p = 0.02 and 0.04, respectively) (**Extended Data** Fig. 2).

Previous CD19 CAR T cell studies in lymphoma demonstrated a significant association between CRS/ICANS and elevated levels of cytokines, including IFNγ, IL-2, IL-6, IL-8, IL-10, IL-15, and TNFα^1,20–22^. We only observed a modest increase of IFNγ and minimal changes for other cytokines in all patients (**Extended Data** Fig. 3). An early increase of IL-15 levels from baseline to T cell infusion day likely reflects the impact of lymphodepleting chemotherapy as previously described^23,24^. There was no difference in the serum concentrations of these cytokines between DL1 and DL2–4 (**Extended Data** Fig. 3).

### Cellular Immunophenotypes of Apheresis and Infused CAR T Products

We observed that preinfusion 1XX CAR T cell products contained a high proportion of CD8^+^ memory T cell subsets including central memory (T_CM_), stem cell memory (T_SCM_) and transitional memory (T_TM_) T cells (Fig. 4b) and low levels of inhibitory checkpoint receptors PD1, LAG3 and TIM3 (**Extended Data** Fig. 4). The infusion products from responders contained a significantly higher proportion of CAR + CD8 + and CD4 + T_CM_ (p < 0.01 and p < 0.05, respectively) and lower proportion of CAR + CD8 + T_TE_ cells compared to non-responders (p < 0.05). (**Extended Data** Fig. 4). Finally, we compared preinfusion product immunophenotypes of 1XX CAR T cells to those of commercial axicabtagene ciloleucel, an archetypal CD19-directed CD28/CD3z CAR. We found 1XX CAR T cells were more likely to express CD127 (IL7R) and less likely to express the inhibitory receptor PD1 (**Extended Data Fig. 5**).

## Discussion

This is the first report of the clinical safety and efficacy of a 1XX CAR. In this phase 1 trial enrolling r/r LBCL patients, we aimed to assess whether 1XX CAR T cells are effective at low doses and associated with low toxicity. We investigated 4 escalating doses of 19(T2)28z-1XX CAR T cells, ranging from 25×10^6^ to 200×10^6^, and expanded the lowest dose (25×10^6^) based on similitude of responses and C_max_ seen at all dose levels. In the 16 patients infused with 25×10^6^ 1XX CAR T cells, we report ORR and CR rates of 88% and 75%, respectively, with only 1 patient experiencing grade 3 CRS and ICANS.

The CR and ORR rates of 75% and 88% observed with the low dose (25×10^6^) 1XX CAR compare favorably to 40–65% CR and 52–83% ORR reported with axicabtagene (2×10^6^ CAR T cells/kg), lisocabtagene (100×10^6^ CAR T cells) and tisagenlecleucel (300×10^6^ CAR T cells) in the 2nd and 3rd line settings^1–4^. The median sum of the product of the diameters (SPD) of 1,430mm^2^ in the study was comparable to those reported in the second line studies of lisocabtagene (median SPD 1,140mm^2^)^6^ and axicabtagene (median SPD 2,123mm^2^)^2^. These outcomes demonstrate the potency of 1XX CAR T cells, which yield equivalent to higher response rates at a dose 4–12 times lower than the conventional doses. Furthermore, the responses observed in the study appear durable. With a median follow-up duration of 24 months, 15 of 20 patients (75%) who achieved CR remain in continuous remission of ≥ 12-month duration with the 1-year EFS rate of 61%, comparable to the estimated 1-year PFS rates of 44–46% reported with the conventional CD19 CARs in both 2nd and ≥ 3rd line settings^1,3,4^.

Our intent to select and expand the lowest effective dose is unique and deviates from typical phase I studies where the maximum tolerated dose is selected for dose expansion. Based on preclinical 1XX data^15^, we purposely designed the study to select a biologically active and clinically effective dose based on early efficacy, safety and response analysis as recently endorsed by the FDA^25^. In addition to reducing toxicity, lower T cell dosages will lead to a shorter manufacturing time, cut down material usage and alleviate supply shortages, thereby increasing overall manufacturing capacity and reducing the cost of goods, and may allow peripheral blood collection to supersede apheresis, lessening logistical barriers to autologous T cell therapy we currently face today. A CAR design requiring lower T cell dosage such as 1XX is also well suited for in vivo T cell engineering wherein delivery to T cells may be limiting.

Low-dose 1XX CAR T cell infusion resulted in only 1 of 16 patients experiencing ≥ grade 3 CRS and ICANS. Notably, the overall incidence of ≥ grade 3 CRS and ICANS was low across all dose levels, including at the highest doses of 100 and 200×10^6^ CAR T cells (4% and 7%, respectively). These rates compare favorably to 6–13% and 21–28% rates of severe CRS and ICANS reported with conventional CD28 containing CD19 CARs such as axicabtagene^1,2^.

Interestingly, the low toxicity profile is obtained with a high affinity CAR. The single-chain variable fragment (scFv) used in our 1XX CAR (**Extended Data Fig. 6**) has high affinity (K_D_ 9.1nM) for CD19, similar to FMC63 (4.5–5.1nM) used in axicabtagene^26,27^ and higher than CAT (14nM) used in obecabtagene autoleucel^28^ or SJ25C1 (27nM) used in our prior studies^8^. Thus, our clinical data demonstrate that a high-affinity binder coupled to the CD28–1XX CAR signaling module yields potent CAR T cells that can be administered at a low dose and achieve disease control with the preserved high proliferative potential of CD28 CAR T cells but with low toxicity. Robust T cell expansion without severe CRS/ICANS is an attractive feature for treating other diseases where higher severe CRS or ICANS rates with conventional CAR T cells require dose reductions or split dose administration in high disease burden patients^8,12,29–31^.

Preinfusion T cell phenotypes can identify high quality cell populations that are associated with T cell fitness and expansion^32,33^. We observed significant skewing toward CD8^+^ T_CM_ and T_SCM_ phenotypes during the manufacturing process, resulting in a relatively high content of less-differentiated T cell subsets in the infusion products, consistent with our preclinical studies^15^. Compared to axicabtagene, which contains the identical CD28 and CD3z domains but unmodified ITAMs, the 1XX CAR yields an infusion product with significantly higher expression of IL7R and lower PD1, which likely contributes to increased CAR T cell persistence as observed in other settings^33–35^. These features of the 1XX signaling module may thus be useful in other lymphoid diseases such as chronic lymphocytic leukemia^33,36^ and in solid tumors as suggested in a preclinical ovarian tumor model^37^.

In conclusion, our data establish that a 1XX CAR with enhanced signaling calibration confers potent anti-tumor efficacy and promotes T_CM_ content in the infusion products, yielding robust response and progression-free survival with moderate toxicity and without requiring infusion of hundreds of millions of CAR T cells as is the case with other CARs. These findings support continued investigation of 1XX CARs targeting CD19 or other antigens in the setting of solid tumors and autoimmune diseases.

## Methods

### Study Design and Patients

This was a phase 1 trial conducted at Memorial Sloan Kettering Cancer Center (MSK) where CAR T cells were manufactured (ClinicalTrials.gov registration: NCT04464200). Eligible patients were ≥ 18 years of age with a diagnosis of r/r LBCL including diffuse large B-cell lymphoma (DLBCL), transformed DLBCL from indolent B-cell lymphoma, or high-grade B cell lymphoma. Patients needed to have refractory disease, defined as progressive or stable disease as the best response to the most recent chemotherapy regimen, disease progression or recurrence in ≤ 12 months of prior autologous stem cell transplant, or relapsed disease after ≥ 2 prior chemoimmunotherapies with at least one containing an anthracycline and CD20 directed therapy. Patients with prior CD19-directed therapy were allowed if CD19 expression was confirmed at screening.

Bridging therapy after apheresis was allowed at the treating physician’s discretion. Positron emission tomography/computerized tomography (PET/CT) scan was obtained at screening and prior to lymphodepleting chemotherapy. Enrolled patients received fludarabine 30mg/m^2^/d and cyclophosphamide 300mg/m^2^/d for 3 days followed by a single infusion of CAR T cells. The trial followed a standard 3 + 3 dose escalation with 4 escalating doses of CAR T cells: 25×10^6^, 50×10^6^, 100×10^6^, and 200×10^6^. The experimental design allowed for expansion cohorts of up to 12 patients to further characterize the safety and efficacy of a specific dose level (DL).

### Study Oversight

The clinical trial was approved by the MSK Institutional Review Board and was conducted in accordance with the Declaration of Helsinki and International Conference on Harmonization guidelines for Good Clinical Practice. Written informed consent was obtained from patients before start of the treatment. All authors had access to the data and were involved in the analysis of the results and vouch for the data and adherence to the protocol.

### Endpoints and Assessments

The primary study objectives were to assess safety and tolerability and determine the recommended dose for an expansion cohort. Secondary endpoints included the rates of best overall response, complete response (CR), progression-free survival (PFS), and evaluation of expansion and persistence of 19(T2)28z-1XX CAR T cells. CRS and ICANS were graded according to the American Society for Transplantation and Cellular Therapy criteria^11^. Disease response was assessed per the Lugano Criteria^38^. All other toxicities were assessed per the Common Terminology Criteria for Adverse Events version 5.0. Disease response was assessed by PET/CT scan at months 1, 3 and 6, and as clinically indicated thereafter.

### Metabolic Tumor Volume Measurements

18F-FDG PET/CT was performed on Discovery 690 and 710 scanners (GE Healthcare, Waukesha, WI). PET emission scans (2–3 min/bed position) and low-dose CT (120–140kV; 80mA) of the skull base to upper thigh were obtained approximately one hour after the intravenous injection of 18F-FDG (444 MBq ± 10%). Blood glucose levels were < 180 mg/dL. Low-dose non-contrast-enhanced CT was used for attenuation correction. A heavy z-axis and Gaussian transaxial filter with 6.4 mm cutoff were used. All scans passed visual quality control. Metabolic tumor volume (MTV) was calculated semi-automatically by a board-certified radiologist using the Beth-Israel PET/CT viewer plugin for FIJI^39^. Lower and upper thresholds for SUV were 4 and 200. The reader had access to clinical data, prior and follow-up imaging and reports.

### Cellular Kinetics

DNA from patient blood samples collected in EDTA tubes were extracted using Promega Maxwell RSC Blood DNA Kit and instrument, and genomic DNA concentration was quantified using Promega Quantus Instrument, following manufacturer’s recommendations. Presence of CAR + cells were quantified and normalized to the endogenous number of diploid albumin copies using Thermo Fisher QuantStudio Real-time PCR systems as previously described^40^. Briefly, duplicate reactions were set up in MicroAmp Optical 96-well reaction plates in 25uL per well containing 1x PCR mix and 100ng of gDNA for each sample, with proper controls and standards. Data Analysis was performed using the QuantStudio real-time PCR software.

### Cell Isolation and Multiparametric Flow Cytometric Analysis

Peripheral blood mononuclear cells (PBMCs) were isolated from EDTA-treated peripheral blood by Ficoll-Paque (Cytiva) density centrifugation, in SepMate tubes (StemCell Technologies), according to the manufacturer’s specifications. Next, cells were washed and resuspended in PBS, then incubated with Human TruStain FcX Fc receptor blocking solution (Biolegend) and Live/DEAD Fixable Blue Dead Cell Stain (Invitrogen) according to the manufacturers’ specifications for 20 minutes at room temperature (RT), protected from light. The cells were washed once in Flow Wash Buffer (FWB; RPMI 1640 no phenol red + 4% FBS + 0.01% sodium-azide) and incubated with the antibody mix for 20 minutes at RT in the dark in the presence of Brilliant Staining Buffer (BD). The cells were washed, resuspended in 0.5% paraformaldehyde/PBS, and immediately acquired using a Cytek Aurora 5L flow cytometer (Cytek). The optimal concentration of all antibodies used in the study was defined by titration. Further information about the antibodies can be found in **Extended Table 5**. Analysis was performed with FlowJo v10.8.1.

### Statistical Analysis

The study included a dose-escalation phase, which utilized a 3 + 3 dose-escalation design, followed by a dose-expansion phase. The definitions of dose-limiting toxicity (DLTs) are outlined in the study protocol (Supplementary Appendix). The DLT window was 28 days following infusion of CAR T cells. Descriptive statistics summarized the adverse events that occurred on study. Kaplan-Meier survival curves estimated both overall survival and EFS from the time of CAR T cell infusion. A Wilcoxon rank-sum test compared metabolic tumor volume, cellular kinetics and cell product immunophenotypes by either response or toxicity categories. A local polynomial smoother was used to summarize serum cytokines over time.

## Figures and Tables

**Table 1.** Baseline Patient and Disease Characteristics

Characteristic	Number (%)

No. of patients	28

Disease type - no. (%)	
DLBCL	17 (61)
HGBCL	4 (14)
Transformed indolent B cell lymphoma	7 (25)

Age	
Median (range) - yr.	65 (48 – 86)
≥65 yr. - no. (%)	13 (46)

Male sex - no. (%)	15 (54)

Prior therapies - no. (%)	
Primary refractory disease	24 (86)
Refractory to last line of therapy pre-apheresis	25 (89)
Prior autologous SCT	3 (11)
Prior CD19 directed therapy	3 (11)

Prior lines of therapy	
Median (range)	1 (1 – 6)
≥2 prior lines of therapy - no. (%)	11 (39)

Genomics*	
Double-hit	7 (30)
TP53 mutated	7 (30)
Double-hit or TP53 mutated	12 (52)

Bridging therapy - no. (%)	25 (89)
Chemotherapy	19 (68)
Radiation	6 (21)

Metabolic tumor volume, median (range) - ml	
At enrollment	51.8 (0.5 – 831.9)
At lymphodepleting chemotherapy**	37.1 (0 – 413.3)

Median tumor burden at lymphodepleting chemotherapy^¶^ (range) - mm^2^	1,430 (0 – 8,611)

LDH > ULN at enrollment - no. (%)	14 (50)
Median LDH (range), U/l	349 (152 – 3,754)

LDH > ULN at lymphodepleting chemotherapy - no. (%)	11 (39)
Median LDH (range), U/l	181 (141 – 1,073)

CRP at lymphodepleting chemotherapy	
Median CRP (range), mg/dL	0.30 (0.07 – 18.70)

Abbreviations: DLBCL, diffuse large B-cell lymphoma; HGBCL, high grade B-cell lymphoma; PMBCL, primary mediastinal B-cell lymphoma; SCT, stem cell transplant; SPD: sum of the products of diameters; LDH: lactate dehydrogenase; ULN, upper limit of normal; CRP, C-reactive protein.

* Available in 23 patients.

** Restaged after bridging (if administered) and immediately prior to lymphodepleting chemotherapy and prior to CAR T cell infusion.

¶ Tumor burden was determined on the basis of the sum of product diameters of the target lesions, according to the Cheson criteria^41^.

**Table 2:** All Adverse Events Regardless of Causality

Number of patients (percent)				
Event	Any Grade	Grade 1	Grade 2	Grade ≥ 3
CRS	24 (86)	11 (39)	12 (43)	1 (4)
ICANS	3 (11)	1 (4)	0	2 (7)
Diarrhea	7 (25)	7 (25)	0	0
Nausea	6 (21)	5 (18)	1 (4)	0
Edema, limbs or generalized	5 (18)	4 (14)	0	1 (4)
Headache	4 (14)	3 (11)	1 (4)	0
Fatigue	4 (14)	4 (14)	0	0
Hypotension	4 (14)	1 (4)	2 (7)	1 (4)
Dizziness	3 (11)	3 (11)	0	0
COVID19	3 (11)	2 (7)	1 (4)	0
Tremor	3 (11)	2 (7)	1 (4)	0
Enterocolitis	3 (11)	0	3 (11)	0
Sinus tachycardia	3 (11)	3 (11)	0	0
Erythema multiforme	2 (7)	1 (4)	1 (4)	0
Floaters	2 (7)	2 (7)	0	0
Peripheral sensory neuropathy	2 (7)	2 (7)	0	0
Cough	2 (7)	2 (7)	0	0
Pain	2 (7)	1 (4)	1 (4)	0
Dysphagia	2 (7)	2 (7)	0	0
Delirium	2 (7)	0	0	2 (7)
Chills	2 (7)	2 (7)	0	0
Myalgia	1 (4)	0	1 (4)	0
Sepsis	1 (4)	0	0	1 (4)
Abdominal pain	1 (4)	1 (4)	0	0
Alopecia	1 (4)	1 (4)	0	0
Anxiety	1 (4)	1 (4)	0	0
Arthralgia	1 (4)	1 (4)	0	0
Atrial fibrillation	1 (4)	0	0	1 (4)
Cystitis	1 (4)	0	1 (4)	0
Constipation	1 (4)	0	1 (4)	0
Dysuria	1 (4)	1 (4)	0	0
Decreased fibrinogen	1 (4)	0	1 (4)	0
Hyperhidrosis	1 (4)	1 (4)	0	0
Muscle weakness, lower limb	1 (4)	1 (4)	0	0
Parainfluenza	1 (4)	1 (4)	0	0
Paresthesia	1 (4)	1 (4)	0	0
Pleural effusion	1 (4)	1 (4)	0	0
Rash	1 (4)	1 (4)	0	0
Rhinorrhea	1 (4)	1 (4)	0	0
Sore throat	1 (4)	1 (4)	0	0
Urinary frequency	1 (4)	1 (4)	0	0
Vomiting	1 (4)	1 (4)	0	0
Weight loss	1 (4)	1 (4)	0	0
Confusion	1 (4)	1 (4)	0	0
Dyspnea	1 (4)	1 (4)	0	0
Fever	1 (4)	1 (4)	0	0
Hypoxia	1 (4)	0	1 (4)	0

Twenty-four patients (86%) experienced CRS. All occurrences of CRS were grade 1 (n = 11, 39%) or 2 (n = 12; 43%), except for 1 grade 3 CRS (4%) (Fig. 2 a). Severity and occurrences of CRS by dose levels are shown in Fig. 2 b. No patient experienced grade 4 or 5 CRS. Median time to CRS onset was 4 days (range, 1–11) and median duration was 4 days (range, 1–9). Seventeen patients (61%) received tocilizumab and 9 (32%) received steroids as treatment for CRS. ICANS was observed in 3 patients: grade 1 (n = 1, 4%) and grade 3 (n = 2, 7%) (Fig. 2 c). No patient experienced grade 4 or 5 ICANS. Median time to ICANS onset was 8 days (range, 7–10 days), and median duration was 9 days (range, 3–10). All 3 patients with ICANS received steroids as treatment with resolution of symptoms.

## References

[1] Neelapu SS, Locke FL, Bartlett NL, Axicabtagene Ciloleucel CAR T-Cell Therapy in Refractory Large B-Cell Lymphoma. New England Journal of Medicine 2017;377(26):2531–2544. DOI: 10.1056/NEJMoa1707447. 29226797 PMC5882485

[2] Locke FL, Miklos DB, Jacobson CA, Axicabtagene Ciloleucel as Second-Line Therapy for Large B-Cell Lymphoma. N Engl J Med 2022;386(7):640–654. DOI: 10.1056/NEJMoa2116133. 34891224

[3] Abramson JS, Palomba ML, Gordon LI, Lisocabtagene maraleucel for patients with relapsed or refractory large B-cell lymphomas (TRANSCEND NHL 001): a multicentre seamless design study. Lancet (London, England) 2020;396(10254):839–852. (In eng). DOI: 10.1016/s0140-6736(20)31366-0. 32888407

[4] Schuster SJ, Bishop MR, Tam CS, Tisagenlecleucel in Adult Relapsed or Refractory Diffuse Large B-Cell Lymphoma. N Engl J Med 2019;380(1):45–56. DOI: 10.1056/NEJMoa1804980. 30501490

[5] Bishop MR, Dickinson M, Purtill D, Second-Line Tisagenlecleucel or Standard Care in Aggressive B-Cell Lymphoma. N Engl J Med 2022;386(7):629–639. DOI: 10.1056/NEJMoa2116596. 34904798

[6] Abramson JS, Solomon SR, Arnason J, Lisocabtagene maraleucel as second-line therapy for large B-cell lymphoma: primary analysis of the phase 3 TRANSFORM study. Blood 2023;141(14):1675–1684. DOI: 10.1182/blood.2022018730. 36542826 PMC10646768

[7] Wang M, Munoz J, Goy A, KTE-X19 CAR T-Cell Therapy in Relapsed or Refractory Mantle-Cell Lymphoma. New England Journal of Medicine 2020;382(14):1331–1342. DOI: 10.1056/NEJMoa1914347. 32242358 PMC7731441

[8] Park JH, Riviere I, Gonen M, Long-Term Follow-up of CD19 CAR Therapy in Acute Lymphoblastic Leukemia. N Engl J Med 2018;378(5):449–459. DOI: 10.1056/NEJMoa1709919. 29385376 PMC6637939

[9] Maude SL, Laetsch TW, Buechner J, Tisagenlecleucel in Children and Young Adults with B-Cell Lymphoblastic Leukemia. N Engl J Med 2018;378(5):439–448. DOI: 10.1056/NEJMoa1709866. 29385370 PMC5996391

[10] Porter DL, Levine BL, Kalos M, Bagg A, June CH. Chimeric antigen receptor-modified T cells in chronic lymphoid leukemia. N Engl J Med 2011;365(8):725–33. DOI: 10.1056/NEJMoa1103849. 21830940 PMC3387277

[11] Lee DW, Santomasso BD, Locke FL, ASTCT Consensus Grading for Cytokine Release Syndrome and Neurologic Toxicity Associated with Immune Effector Cells. Biology of blood and marrow transplantation : journal of the American Society for Blood and Marrow Transplantation 2019;25(4):625–638. (In eng). DOI: 10.1016/j.bbmt.2018.12.758. PMC12180426 30592986

[12] Shah BD, Ghobadi A, Oluwole OO, KTE-X19 for relapsed or refractory adult B-cell acute lymphoblastic leukaemia: phase 2 results of the single-arm, open-label, multicentre ZUMA-3 study. Lancet (London, England) 2021;398(10299):491–502. DOI: 10.1016/S0140-6736(21)01222-8. 34097852 PMC11613962

[13] Cappell KM, Kochenderfer JN. A comparison of chimeric antigen receptors containing CD28 versus 4–1BB costimulatory domains. Nat Rev Clin Oncol 2021;18(11):715–727. DOI: 10.1038/s41571-021-00530-z. 34230645

[14] Globerson Levin A, Riviere I, Eshhar Z, Sadelain M. CAR T cells: Building on the CD19 paradigm. Eur J Immunol 2021;51(9):2151–2163. DOI: 10.1002/eji.202049064. 34196410 PMC9392049

[15] Feucht J, Sun J, Eyquem J, Calibration of CAR activation potential directs alternative T cell fates and therapeutic potency. Nat Med 2019;25(1):82–88. DOI: 10.1038/s41591-018-0290-5. 30559421 PMC6532069

[16] Maher J, Brentjens RJ, Gunset G, Riviere I, Sadelain M. Human T-lymphocyte cytotoxicity and proliferation directed by a single chimeric TCRzeta /CD28 receptor. Nat Biotechnol 2002;20(1):70–5. DOI: 10.1038/nbt0102-70. 11753365

[17] Morris EC, Neelapu SS, Giavridis T, Sadelain M. Cytokine release syndrome and associated neurotoxicity in cancer immunotherapy. Nat Rev Immunol 2022;22(2):85–96. DOI: 10.1038/s41577-021-00547-6. 34002066 PMC8127450

[18] Fajgenbaum DC, June CH. Cytokine Storm. N Engl J Med 2020;383(23):2255–2273. DOI: 10.1056/NEJMra2026131. 33264547 PMC7727315

[19] Siddiqi T, Maloney DG, Kenderian SS, Lisocabtagene maraleucel in chronic lymphocytic leukaemia and small lymphocytic lymphoma (TRANSCEND CLL 004): a multicentre, open-label, single-arm, phase 1–2 study. Lancet (London, England) 2023;402(10402):641–654. DOI: 10.1016/S0140-6736(23)01052-8. 37295445 PMC11753452

[20] Santomasso BD, Park JH, Salloum D, Clinical and Biological Correlates of Neurotoxicity Associated with CAR T-cell Therapy in Patients with B-cell Acute Lymphoblastic Leukemia. Cancer discovery 2018;8(8):958–971. (In eng). DOI: 10.1158/2159-8290.Cd-17-1319. 29880584 PMC6385599

[21] Gust J, Hay KA, Hanafi L-A, Endothelial Activation and Blood–Brain Barrier Disruption in Neurotoxicity after Adoptive Immunotherapy with CD19 CAR-T Cells. Cancer discovery 2017;7(12):1404–1419. DOI: 10.1158/2159-8290.CD-17-0698. 29025771 PMC5718945

[22] Topp MS, van Meerten T, Houot R, Earlier corticosteroid use for adverse event management in patients receiving axicabtagene ciloleucel for large B-cell lymphoma. British journal of haematology 2021;195(3):388–398. (In eng). DOI: 10.1111/bjh.17673. 34590303 PMC9293158

[23] Dudley ME, Wunderlich JR, Yang JC, Adoptive cell transfer therapy following non-myeloablative but lymphodepleting chemotherapy for the treatment of patients with refractory metastatic melanoma. J Clin Oncol 2005;23(10):2346–57. DOI: 10.1200/JCO.2005.00.240. 15800326 PMC1475951

[24] Anthony SM, Rivas SC, Colpitts SL, Howard ME, Stonier SW, Schluns KS. Inflammatory Signals Regulate IL-15 in Response to Lymphodepletion. J Immunol 2016;196(11):4544–52. DOI: 10.4049/jimmunol.1600219. 27183627 PMC4875792

[25] Shah M, Rahman A, Theoret MR, Pazdur R. The Drug-Dosing Conundrum in Oncology - When Less Is More. N Engl J Med 2021;385(16):1445–1447. DOI: 10.1056/NEJMp2109826. 34623789

[26] He C, Mansilla-Soto J, Khanra N, CD19 CAR antigen engagement mechanisms and affinity tuning. Sci Immunol 2023;8(81):eadf1426. DOI: 10.1126/sciimmunol.adf1426. 36867678 PMC10228544

[27] Seigner J, Zajc CU, Dotsch S, Solving the mystery of the FMC63-CD19 affinity. Sci Rep 2023;13(1):23024. DOI: 10.1038/s41598-023-48528-0. 38155191 PMC10754921

[28] Ghorashian S, Kramer AM, Onuoha S, Enhanced CAR T cell expansion and prolonged persistence in pediatric patients with ALL treated with a low-affinity CD19 CAR. Nat Med 2019;25(9):1408–1414. DOI: 10.1038/s41591-019-0549-5. 31477906

[29] Frey NV, Shaw PA, Hexner EO, Optimizing Chimeric Antigen Receptor T-Cell Therapy for Adults With Acute Lymphoblastic Leukemia. J Clin Oncol 2020;38(5):415–422. DOI: 10.1200/JCO.19.01892. 31815579 PMC8312030

[30] Munshi NC, Anderson LD, Jr., Shah N, Idecabtagene Vicleucel in Relapsed and Refractory Multiple Myeloma. N Engl J Med 2021;384(8):705–716. DOI: 10.1056/NEJMoa2024850. 33626253

[31] Raje N, Berdeja J, Lin Y, Anti-BCMA CAR T-Cell Therapy bb2121 in Relapsed or Refractory Multiple Myeloma. N Engl J Med 2019;380(18):1726–1737. DOI: 10.1056/NEJMoa1817226. 31042825 PMC8202968

[32] Locke FL, Rossi JM, Neelapu SS, Tumor burden, inflammation, and product attributes determine outcomes of axicabtagene ciloleucel in large B-cell lymphoma. Blood Adv 2020;4(19):4898–4911. DOI: 10.1182/bloodadvances.2020002394. 33035333 PMC7556133

[33] Fraietta JA, Lacey SF, Orlando EJ, Determinants of response and resistance to CD19 chimeric antigen receptor (CAR) T cell therapy of chronic lymphocytic leukemia. Nat Med 2018;24(5):563–571. DOI: 10.1038/s41591-018-0010-1. 29713085 PMC6117613

[34] Sommermeyer D, Hudecek M, Kosasih PL, Gogishvili T, Maloney DG, Turtle CJ, Riddell SR. Chimeric antigen receptor-modified T cells derived from defined CD8+ and CD4+ subsets confer superior antitumor reactivity in vivo. Leukemia 2016;30(2):492–500. DOI: 10.1038/leu.2015.247. 26369987 PMC4746098

[35] Deng Q, Han G, Puebla-Osorio N, Characteristics of anti-CD19 CAR T cell infusion products associated with efficacy and toxicity in patients with large B cell lymphomas. Nat Med 2020;26(12):1878–1887. DOI: 10.1038/s41591-020-1061-7. 33020644 PMC8446909

[36] Siddiqi T, Soumerai JD, Dorritie KA, Phase 1 TRANSCEND CLL 004 study of lisocabtagene maraleucel in patients with relapsed/refractory CLL or SLL. Blood 2022;139(12):1794–1806. DOI: 10.1182/blood.2021011895. 34699592 PMC10652916

[37] Schoutrop E, Poiret T, El-Serafi I, Tuned activation of MSLN-CAR T cells induces superior antitumor responses in ovarian cancer models. J Immunother Cancer 2023;11(2). DOI: 10.1136/jitc-2022-005691. PMC9906404 36746513

[38] Cheson BD, Fisher RI, Barrington SF, Cavalli F, Schwartz LH, Zucca E, Lister TA. Recommendations for initial evaluation, staging, and response assessment of Hodgkin and non-Hodgkin lymphoma: the Lugano classification. J Clin Oncol 2014;32(27):3059–68. (In eng). DOI: 10.1200/jco.2013.54.8800. 25113753 PMC4979083

[39] Kanoun S, Tal I, Berriolo-Riedinger A, Influence of Software Tool and Methodological Aspects of Total Metabolic Tumor Volume Calculation on Baseline [18F]FDG PET to Predict Survival in Hodgkin Lymphoma. PLoS One 2015;10(10):e0140830. DOI: 10.1371/journal.pone.0140830. 26473950 PMC4608733

[40] Hollyman D, Stefanski J, Przybylowski M, Manufacturing validation of biologically functional T cells targeted to CD19 antigen for autologous adoptive cell therapy. J Immunother 2009;32(2):169–80. DOI: 10.1097/CJI.0b013e318194a6e8. 19238016 PMC2683970

[41] Cheson BD, Pfistner B, Juweid ME, Revised response criteria for malignant lymphoma. J Clin Oncol 2007;25(5):579–86. DOI: 10.1200/JCO.2006.09.2403. 17242396

